# Clinical Feasibility of Continuously Monitored Data for Heart Rate, Physical Activity, and Sleeping by Wearable Activity Trackers in Patients with Thyrotoxicosis: Protocol for a Prospective Longitudinal Observational Study

**DOI:** 10.2196/resprot.8119

**Published:** 2018-02-21

**Authors:** Jie-Eun Lee, Dong Hwa Lee, Tae Jung Oh, Kyoung Min Kim, Sung Hee Choi, Soo Lim, Young Joo Park, Do Joon Park, Hak Chul Jang, Jae Hoon Moon

**Affiliations:** ^1^ Department of Internal Medicine, Seoul National University Healthcare System Gangnam Center Seoul Republic Of Korea; ^2^ Department of Internal Medicine, Seoul National University Bundang Hospital Gyeonggi-do Republic Of Korea; ^3^ Department of Internal Medicine, Seoul National University Hospital Seoul Republic Of Korea

**Keywords:** activity tracker, pulse rate, thyrotoxicosis, hyperthyroidism, Graves’ disease

## Abstract

**Background:**

Thyrotoxicosis is a common disease caused by an excess of thyroid hormones. The prevalence of thyrotoxicosis about 2% and 70-90% of thyrotoxicosis cases are caused by Graves' disease, an autoimmune disease, which has a high recurrence rate when treated with antithyroid drugs such as methimazole or propylthiouracil. The clinical symptoms and signs of thyrotoxicosis include palpitation, weight loss, restlessness, and difficulty sleeping. Although these clinical changes in thyrotoxicosis can be detected by currently available wearable activity trackers, there have been few trials of the clinical application of wearable devices in patients with thyrotoxicosis.

**Objective:**

The aim of this study is to investigate the clinical applicability of wearable device-generated data to the management of thyrotoxicosis. We are analyzing continuously monitored data for heart rate, physical activity, and sleep in patients with thyrotoxicosis during their clinical course after treatment.

**Methods:**

Thirty thyrotoxic patients and 10 control subjects were enrolled in this study at Seoul National University Bundang Hospital. Heart rate, physical activity, and sleep are being monitored using a Fitbit Charge HR or Fitbit Charge 2. Clinical data including anthropometric measures, thyroid function test, and hyperthyroidism symptom scale are recorded.

**Results:**

Study enrollment began in December 2016, and the intervention and follow-up phases are ongoing. The results of the data analysis are expected to be available by September 2017.

**Conclusions:**

This study will provide a foundational feasibility trial of the clinical applications of biosignal measurements to the differential diagnosis, prediction of clinical course, early detection of recurrence, and treatment in patients with thyrotoxicosis.

**Trial Registration:**

ClinicalTrials.gov NCT03009357; https://clinicaltrials.gov/ct2/show/NCT03009357 (Archived by WebCite at http://www.webcitation.org/6wh4MWPm2)

## Introduction

Thyrotoxicosis is clinical term that refers collectively to all symptoms that occur when an excess of thyroid hormones (free thyroxine [T4] and/or free triiodothyronine [T3]) is supplied to the peripheral tissues. The term “hyperthyroidism” is used in a similar way and refers to thyrotoxicosis caused by excessive production and secretion of thyroid hormone from the thyroid gland. Thyrotoxicosis is caused by hyperthyroidism, but it can occur without hyperthyroidism. Graves’ disease is an organ-specific autoimmune disease in which autoantibodies to thyroid-stimulating hormone receptors stimulate the thyroid gland, which causes hyperthyroidism. The prevalence of thyrotoxicosis is about 2%, and 70-90% of cases are caused by Graves’ disease, although the rates and causes vary between different geographic areas [1,2]. The incidence and prevalence rates of thyrotoxicosis have not been reported in Korea. However, among thyrotoxic patients treated in the thyroid clinic of Seoul National University Hospital, the rates of Graves’s disease and thyroiditis were reported to be 82.7% and 16.8%, respectively [3]. Graves’ disease is an autoimmune disease. There are 3 options for treating patients with this disease: antithyroid drugs (ATDs), radioactive iodine ablation, and surgery. The choice of treatment for Graves’s disease differs between geographical regions. Radioactive iodine therapy is frequently used as the first-line therapy in North America [4]. In Europe and Asia, ATDs such as methimazole, propylthiouracil are preferred as the primary treatment [5,6]. A drawback of ATD therapy is the high rate of relapse of hyperthyroidism after the drug has been discontinued. Relapse is more frequent in the first year than in subsequent years, particularly in the first 6 months after stopping the medication [7]. The risk of recurrence varies greatly between patients but is estimated to be 50-55% according to a Cochrane review of 26 randomized clinical trials [8]. Patients with recurrent Graves’ disease often wait a considerable time after recurrence to visit the clinic and often present with aggravated symptoms. Therefore, having a simple effective monitoring tool for evaluating disease status would be helpful for monitoring patients with Graves’ disease after they stop taking an ATD.

Excess thyroid hormone affects many different organ systems. The clinical symptoms signs are fatigue, anxiety, palpitations, sweating, heat intolerance, anxiety, disturbed sleep, and weight loss [9]. These clinical manifestations are relatively nonspecific and can vary depending on several factors, such as the patient’s age, sex, comorbidities, and duration and cause of the disease [10,11]. The variety of nonspecific symptoms and signs makes it difficult to diagnose or assess the disease status based on conventionally obtained information about symptoms and signs. Therefore, thyrotoxicosis is diagnosed by blood tests showing increased thyroid hormone levels. The diagnosis is often delayed because patients do not recognize their symptoms as those of thyroid dysfunction and visit a clinic only when the symptoms become severe.

Wearable activity trackers have grown in popularity over the past few years. The American College of Sports Medicine survey of fitness trends reported that wearable technology was the top-rated trend for 2016 [12]. It was projected that about 32 million wearable activity trackers will have been sold by the end of 2016, and forecasts indicate that sales of these devices will exceed 82 million by 2019 [13]. These devices are typically worn on the wrist or hip and provide information about physical activity measures such as steps taken, horizontal and vertical distances moved, sleep pattern, and even heart rate. Many newer wearable devices use photoplethysmography to monitor heart rate by measuring the differential reflection of light from the skin based on the pulsatility of superficial blood vessels [14]. Several studies have been published on the accuracy of these wrist-worn heart rate monitors and have shown that they are accurate for measuring heart rate in the resting state [15-17]. Therefore, it is now possible to analyze more detailed, precise, and continuously collected “high-definition data” of the changes in heart rate, physical activity, and sleep patterns. This may allow the identification of key symptoms and signs of thyrotoxicosis, which can be nonspecific and difficult to detect by only conventional on-site history taking or physical examination [18]. However, few trials have examined the clinical applicability of wearable devices in monitoring patients with thyrotoxicosis.

The aim of this trial is to investigate whether changes in symptoms and signs of thyrotoxicosis, such as heart rate, physical activity, and sleep pattern, can be monitored by wearable devices during the course of thyrotoxicosis and whether these have clinical applications.

## Methods

### Study Setting

This is a single-centered, prospective observational study to analyze the changes in heart rate, physical activity, and sleep patterns monitored by commercially available wearable devices during the clinical course of thyrotoxicosis. Subjects have been recruited from the outpatient clinic of the endocrinology department at Seoul National University Bundang Hospital (SNUBH). Among those with newly diagnosed or recurrent thyrotoxicosis, we have included 30 patients who met the inclusion criteria. Ten healthy age- and sex-matched volunteers were also included as a control group.

**Table 1:** Inclusion and exclusion criteria for patient and control groups.

Group	Inclusion criteria	Exclusion criteria
Patients	Aged 15-60 years Diagnosed with newly developed or recurrent thyrotoxicosis Who can use a wearable device and smartphone apps Planned to be treated with ATDs if affected by Graves’ disease	A history of thyrotoxic periodic paralysis Thyrotoxicosis caused by toxic nodular goiter Taking medications that can affect heart rate (except short-acting beta-blockers prescribed to relieve thyrotoxic symptoms)
Controls	Aged 15-60 years Confirmed euthyroid state by TFT Who can use a wearable device and smartphone apps	A history of thyroid disease or taking thyroid hormone or an ATD Taking medications that can affect heart rate

^a^ATD: antithyroid drug

^b^TFT: thyroid function test

### Inclusion and Exclusion Criteria

The inclusion criteria for enrolment are listed in Table 1. We have included patients and controls aged 15-60 years because this age range is generally expected to be able to use wearable devices, smartphones, and apps. Patients were excluded from the study if they have a history of thyrotoxic periodic paralysis because paralysis itself can interfere with daily activities. Patients with thyrotoxicosis caused by toxic nodular goiter were also excluded because this condition needs treatment options, such as surgery or radioactive iodine therapy, and gradual improvement is not observed after these treatments. Prescription of beta-blockers to the patients is the usual treatment for the symptoms of thyrotoxicosis. Therefore, we prescribed short-acting beta-blockers to symptomatic patients and recorded their dosing times to minimize the impact on the study.

The healthy control group included euthyroid people who have no history of thyroid disease. We recruited the control group from SNUBH staff and confirmed that they had no history of thyroid disease and no abnormal findings or medications that can affect heart rate through their medical history, which included employee health examinations and an interview.

When patients or controls visited the clinic and agreed to participate in this study, we explained how to use and manage the wearable device (eg, synchronization, charging, basic settings), download and install smartphone apps, and create an account. We then check that they can use and manage their devices by letting them repeat all processes mentioned above.

### Protocol

**Figure 1:**
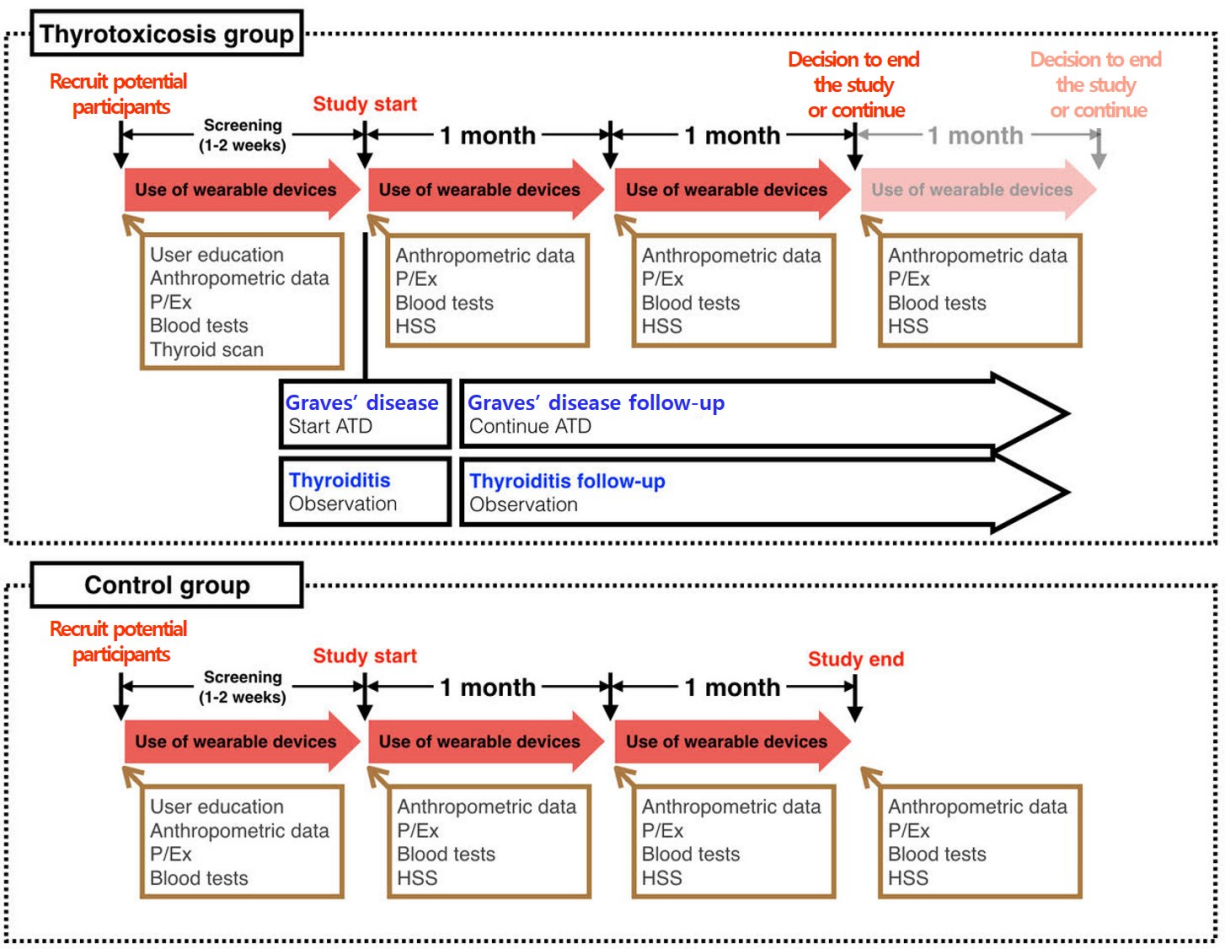
Study design and flow.Blood tests include a thyroid function test, serum levels of antithyroid-stimulating hormone receptor antibody, and other biochemical tests. Tc-99m is used in the thyroid scan. P/Ex, physical examination; HSS, hyperthyroidism symptom scale; ATD, antithyroid drug.

This protocol has been approved by the SNUBH Institutional Review Board (IRB #: B-1609-363-004) and is registered at ClinicalTrials.gov (trial registration number NCT03009357). The study design is displayed in Figure 1. All patients participating in the study were informed of the study and signed a written consent form.

Blood tests include a thyroid function test, serum levels of antithyroid-stimulating hormone receptor antibody, and other biochemical tests. Tc-99m is used in the thyroid scan. P/Ex, physical examination; HSS, hyperthyroidism symptom scale; ATD, antithyroid drug.

The potential candidates of this study are patients with newly detected or recurrent thyrotoxicosis who meet the eligibility criteria. They are provided with a device and given brief on-site instructions for using the device and app, which is installed on their own smartphone. They are instructed to wear the device as much as possible throughout the day including when asleep. We also explain that if they do not wear the device or sync to the app for more than 5 days in a row, they will be excluded from the study because of poor compliance. After 1 to 2 weeks of screening period, the patients visit the clinic to confirm the results of the thyroid function test (TFT) and other tests to distinguish the cause (eg, autoantibodies, thyroid scan) and to start treatment according to the cause of thyrotoxicosis. At this point, patients are finally enrolled, and the study period starts.

Patients with Graves’ disease are prescribed a specific dose of an ATD as decided by the physician. Patients with transient thyrotoxicosis caused by thyroiditis are provided reassurance that the symptoms and signs are benign and self-limited. Patients taking a beta-blocker for control of symptoms, such as palpitations and tremor, are advised to take the drug when the symptoms are severe and to inform the investigator the dose and timing of the drug, which are recorded in the care report form (CRF). Regardless of the cause of thyrotoxicosis, all patients are being followed up once a month, when they undergo blood tests and a physical examination, complete the hyperthyroidism symptom scale (HSS), and have their anthropometric parameters measured. The ATD dose is adjusted as necessary. The study will end after the third visit for each patient. At the discretion of the attending physician and if the patient agrees, the duration of this observational study may be extended if thyroid function is not fully restored. Additional consent for extension of the study period is not necessary because it is already specified in the initial consent form.

Healthy adults have been recruited as a control group through an official announcement in SNUBH. The controls visit the hospital on the same schedule as the patients with thyrotoxicosis. They are provided the same instructions as the patients about using the device and apps installed on their own smartphone, and the importance of compliance. They also undergo the same blood tests as the patients and are instructed to inform the investigator if there is any change in their medication; this information is recorded in the CRF.

After the research process is completed, the subjects who were not excluded from the study during the study observation period will be allowed to keep the device.

### Hyperthyroidism Symptom Scale

To assess the clinical status of hyperthyroidism, the endocrinologist in charge of this study evaluated the patients using the HSS [19]. This scoring system has been reported to reflect the status of thyroid function well [20,21]. This allows us to compare the relationships between the data from the wearable devices and TFTs with those between the HSS and thyroid function. The HSS is a 10-item scale that rates symptoms and signs of thyrotoxicosis; the information needed to rate each item is obtained through history taking and physical examination. Among the 10 items of the HSS, those corresponding to nervousness, sweating, heat intolerance, hyperactivity, weakness, diarrhea, appetite, and assessment of daily function were evaluated through history taking, and tremor and hyperdynamic precordium were evaluated by physical examination. The item scores are totaled to obtain the overall score, which can range from 0 to 40 points.

### Wearable Devices and Apps

We are using the Fitbit charge HR or Fitbit charge 2 (Fitbit, San Francisco, CA, USA) and Fitbit apps for iOS or Android. The firmware version of this app is currently 18.122 and this latest version has been maintained continuously over the observation period.

During the study period and with the patient’s consent, each participant’s account information from the Fitbit app, including identification number and password, are shared with researchers, which allows researchers to access the account of Fitbit website [22] and monitoring of sync status between the Fitbit app and the individual patient’s Fitbit tracking device. At the end of the study, we will separate the account from the device and allow each participant to have their own account.

### Anthropometric and Biochemical Measurements

Height and weight are measured with the subject in light clothing and without shoes to the nearest 0.1 cm and 0.1 kg, respectively. Body mass index is calculated as the ratio of weight and to the square of height (expressed in kilograms per square meter). Blood pressure and heart rate were measured on the right arm with the subject in a seated position. Serum levels of blood urea nitrogen, creatinine and glucose were measured by automated standard laboratory methods (Hitachi 747; Hitachi, Tokyo, Japan). Serum total protein, albumin, total bilirubin, alkaline phosphatase, aspartate aminotransferase, and alanine aminotransferase were measured with an autoanalyzer (TBA-200FR; Toshiba, Tokyo, Japan). For TFT, concentrations of serum T4 (DiaSorin, Saluggia, Italy) and thyroid-stimulating hormone (TSH; CIS Bio International, Gif-sur-Yvette, France) were measured using immunoradiometric assays. The free T4 assay had an analytical sensitivity of 0.05 ng/dl, while that for TSH had an analytical sensitivity of 0.04 mIU/l and a functional sensitivity of 0.07 mIU/l. The reference ranges for free T4 and TSH were 0.89-1.79 ng/dl and 0.3-4.0 mIU/l, respectively. Thyrotoxicosis was defined based on the results of the TFT: that is, overt thyrotoxicosis was defined as high free T4 and low TSH, and subclinical thyrotoxicosis as normal free T4 and low TSH. All subjects were examined for the presence of anti-TSH receptor antibody by radioimmunoassay (Cis Bio International) and the cutoff for positivity was >1.0 U/ml.

### Outcome Measures

The primary goal of this study is to verify the changes in heart rate, physical activity, and sleep-related values generated from wearable devices during the clinical course of thyrotoxicosis. We will compare these device-generated data with conventionally evaluated symptoms and signs and analyze the relationships between each parameter and disease status.

#### Heart Rate

Using the heart rate data recorded by the device and provided by the Fitbit database mentioned below, we will analyze all summary and detailed heart rate data including resting heart rate for the study duration.

#### Physical Activity

We will analyze all summary and detailed data for physical activity, including total steps per day and total moving distance per day in both the horizontal and vertical directions.

#### Sleep

We will analyze the sleep data including total time asleep, total number and time of awakening, and sleep efficiency, which is calculated as the total time asleep divided by the total time in bed. We will also assess the data for “minutes to fall asleep” using the app mentioned above.

### Data Collection

**Figure 2:**
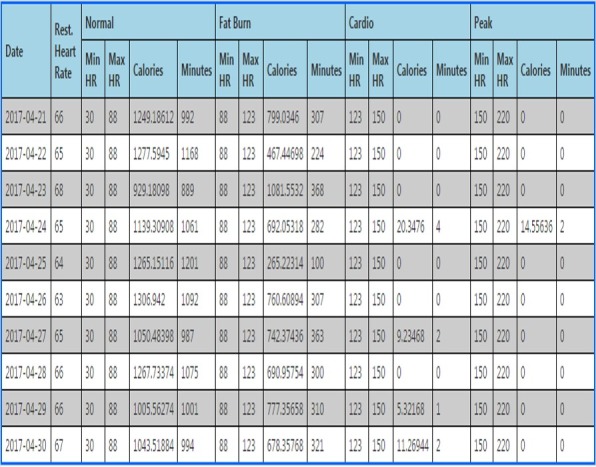
Examples of downloaded heart rate data. Summary.

**Figure 3:**
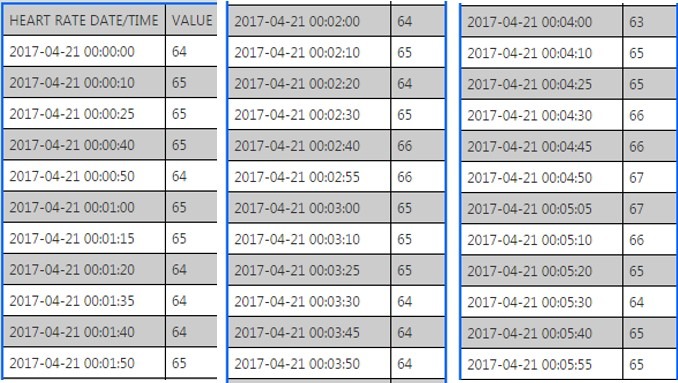
Examples of downloaded heart rate data. Intraday.

We can download interday summary and intraday detailed data for heart rate, physical activity, and sleep from the Fitbit database in JSON format using an app programming interface which is provided by Fitbit [23]. An example of the downloaded data is shown in Figures 2 and 3.

### Data Analysis

To compare variables between the patient and control groups, we will use the Mann–Whitney test or Student’s *t* test for continuous variables and the chi-square test for categorical variables. Repeated-measures ANOVA or the Friedman test will be used to analyze the changes in TFT, blood pressure, heart rate, and physical indices measured by wearable devices. The relationships between disease status and heart rate, physical activity, and sleep will be derived from the linear model of generalized estimating equation (GEE) analyses. Models to prediction thyrotoxicosis will incorporate variables related to heart rate, physical activity, and sleep derived from the binary logistic model of the GEE analyses. Odds ratios with 95% confidence intervals will be computed per standard deviation increase in these variables in relation to the risk of thyrotoxicosis. All statistical analyses will be performed using IBM SPSS Statistics (version 20.0; IBM Corp, Armonk, NY, USA) and R (version 3.3.3; The R Foundation for Statistical Computing, Vienna, Austria).

## Results

Study enrollment began in December 2016. The recruitment of the patient group was completed, and two of the total 30 patients were excluded. One discontinued the study because of a skin reaction to the device (Fitbit Charge HR) on the wrist, and one patient was dropped from the study because of poor compliance in using the device. The remaining 28 patients and all controls completed the study. The data collection process was completed by August 2017. We expect to report the data analysis results in early 2018.

## Discussion

In this prospective, observational study, we will investigate the clinical applicability of wearable device-generated data to the management of thyrotoxicosis by analyzing continuously monitored data for heart rate, physical activity, and sleep for patients with thyrotoxicosis during their clinical course after treatment.

Monitoring heart rate in patients with thyrotoxicosis is required because palpitations or tachycardia are among the most common symptoms of thyrotoxicosis [24]. However, heart rate measurement only in the clinic is not enough to obtain valuable information about the disease status because heart rate can be affected by various physical, mental, and circumstantial factors. Therefore, continuous monitoring of heart rate and physical activity using wearable devices can generate much more precise data about heart rate. For example, conventionally, resting heart rate is measured after at least 10 minutes of lying position and it can vary with patients’ emotion, body position, or air temperature. Wearable devices continuously measure patients’ heart rate and activity and calculate resting heart rate according to the algorithm they have. Generally, resting heart rate of a specific day can be calculated from all heart rate data which are recorded in the time windows with no physical activity during at least 10 minutes (the algorithm can weight to the heart rate data recorded in sleep time). Although user’s heart rate still can be affected by other factors mentioned above, this method based on “high-definition data” can generate more consistent and precise data.

Abnormalities in sleep regulation often occur in patients with thyrotoxicosis [25]. However, there are few reports that specifically address sleep problem related to thyrotoxicosis. Sridhar et al performed a retrospective review of medical records to assess the quality of sleep in patients with thyrotoxicosis and found that thyrotoxicosis was associated principally with difficulty falling asleep and maintaining sleep, and that these were related to hyperkinetic features (tremor, appetite change, bowel disturbances) [26]. In our study, we will analyze various indexes related to sleep prospectively according to later changes in thyroid function, which will provide valuable information on the relationship between the quality of sleep and thyroid function.

Patients with hyperthyroidism exhibit a characteristic resting tremor and self-reported increase in voluntary physical activity, heat intolerance, and weight loss [27]. Studies on physical activity and hyperthyroidism in humans have been rarely reported. A study of rats focused on whether physical activity increases because of hyperthyroidism and found that the thyroid hormone excess was associated with increased voluntary physical activity [28]. There are many ways to measure physical activity in humans, such as accelerometers, pedometers, heart rate monitors, and armbands, but this is quite complex [29]. It is also important to choose the most appropriate method for obtaining the data we intend to collect because none of these methods alone can assess all facets of physical activity. Moreover, because thyrotoxicosis can increase heart rate, assessing physical activity using only heart rate monitoring is not suitable for this study. Therefore, wearable devices with an accelerometer, global positioning system, and heart rate sensor in one device will provide more accurate physical activity data.

The major strength of this study is that it is the first study to monitor heart rate, physical activity, and sleep throughout the day using commercially available wearable devices in patients with thyrotoxicosis. This study also has some important clinical implications. We expect that the data derived from the continuous monitoring of these clinical parameters will be useful for the differential diagnosis of thyrotoxicosis. The two most common causes of thyrotoxicosis are Graves’ disease and thyroiditis. Although the clinical symptoms and signs tend to be more abrupt in patients with thyroiditis, clinically it is difficult to distinguish these through conventional interviews and physical examination. Therefore, continuously collected “high-definition data” should help to differentiate the clinical presentation according to the cause of thyrotoxicosis. If so, this will save time and cost in not having to perform the biochemical and radiological tests currently needed to make the differential diagnosis.

We also expect that these high-definition data may be useful for predicting the therapeutic response and clinical course. After starting the medication, patients must repeat blood testing for thyroid hormone levels every 1 or 2 months. If the dose of medication is insufficient or the disease is unresponsive to the medical treatment, the patient must endure the clinical symptoms until the next visit. Ideally, more frequent tests will provide more detailed information about the clinical course, but more frequent tests also inconvenience the patient and add to the cost. Being able to monitor the clinical course using biosignals from wearable devices may provide patients with faster and more accurate interventions during the treatment and follow-up process. In addition, if these detailed clinical parameters accurately reflect the changes in thyroid function, we expect that the recurrence of thyrotoxicosis may be detected earlier. We hope that the results of this study will allow us to develop apps to calculate the real-time risk of thyrotoxicosis for patients who discontinue their medication and therefore encourage the patient to attend the clinic promptly to receive the diagnosis of and treatment for recurrent thyrotoxicosis.

There are some limitations, which should be considered when interpreting the results. First, to control their symptoms, about 50% of the patients with thyrotoxicosis have been prescribed the nonselective beta-blocker propranolol with a relatively short duration of action. However, patients in the current study who have been prescribed medications have so far reported that they have not taken it continuously for more than 1 day; the drug’s maximum effects appear 60-90 minutes after oral administration [30]. The heart rate will be monitored constantly even when it is not expected to be affected by the propranolol taken. Therefore, the effect of beta-blockade on the primary outcome of this study should be minimized by recording the date and time of taking each propranolol dose. In addition, the small sample size and single study site will provide useful results, but the study will need to be replicated with more participants and study sites.

The technologies to measure and analyze biosignals have not been developed fully, but have been extended from specific medical uses, such as in intensive care units or surgical monitoring, to everyday life. This evolution of technologies will eventually allow for easy, effective, and continuous monitoring of chronic diseases. Thyrotoxicosis changes various biosignals including heart rate and physical activity through the effect of excessive thyroid hormone. Currently available commercial wearable devices are expected to detect these changes in biosignals in thyrotoxic patients. We expect our study to provide clinical evidence of the usefulness of wearable devices for managing thyrotoxicosis.

## Abbreviations

ATD: antithyroid drug
CRF: case report form
GEE: generalized estimating equation
HSS: hyperthyroidism symptom scale
SNUBH: Seoul National University Bundang Hospital
T3: triiodothyronine
T4: thyroxine
TFT: thyroid function test
TSH: thyroid hormone stimulating hormone
